# Prenatal Famine and Genetic Variation Are Independently and Additively Associated with DNA Methylation at Regulatory Loci within *IGF2*/*H19*

**DOI:** 10.1371/journal.pone.0037933

**Published:** 2012-05-30

**Authors:** Elmar W. Tobi, P. Eline Slagboom, Jenny van Dongen, Dennis Kremer, Aryeh D. Stein, Hein Putter, Bastiaan T. Heijmans, L. H. Lumey

**Affiliations:** 1 Molecular Epidemiology, Leiden University Medical Center, Leiden, The Netherlands; 2 Medical Statistics, Leiden University Medical Center, Leiden, The Netherlands; 3 The Netherlands consortium for Healthy Ageing, Leiden University Medical Center, Leiden, The Netherlands; 4 Biological Psychology, VU University Amsterdam, Amsterdam, The Netherlands; 5 Hubert Department of Global Health, Rollins School of Public Health, Emory University, Atlanta, Georgia, United States of America; 6 Department of Epidemiology, Mailman School of Public Health, Columbia University, New York, New York, United States of America; Deutsches Krebsforschungszentrum, Germany

## Abstract

Both the early environment and genetic variation may affect DNA methylation, which is one of the major molecular marks of the epigenome. The combined effect of these factors on a well-defined locus has not been studied to date. We evaluated the association of periconceptional exposure to the Dutch Famine of 1944–45, as an example of an early environmental exposure, and single nucleotide polymorphisms covering the genetic variation (tagging SNPs) with DNA methylation at the imprinted *IGF2/H19* region, a model for an epigenetically regulated genomic region. DNA methylation was measured at five differentially methylated regions (DMRs) that regulate the imprinted status of the *IGF2*/*H19* region. Small but consistent differences in DNA methylation were observed comparing 60 individuals with periconceptional famine exposure with unexposed same-sex siblings at all *IGF2* DMRs (P_BH_<0.05 after adjustment for multiple testing), but not at the *H19* DMR. *IGF2* DMR0 methylation was associated with *IGF2* SNP rs2239681 (P_BH_ = 0.027) and *INS* promoter methylation with *INS* SNPs, including rs689, which tags the *INS* VNTR, suggesting a mechanism for the reported effect of the VNTR on *INS* expression (P_BH_ = 3.4×10^−3^). Prenatal famine and genetic variation showed similar associations with *IGF2*/*H19* methylation and their contributions were additive. They were small in absolute terms (<3%), but on average 0.5 standard deviations relative to the variation in the population. Our analyses suggest that environmental and genetic factors could have independent and additive similarly sized effects on DNA methylation at the same regulatory site.

## Introduction

The epigenome consists of inter-related layers of molecular marks on the DNA that represent non-genetic, but stable and mitotically heritable information determining the gene-expression potential of a genomic region [1]. Studies in animal models show that environmental factors during early development can cause persistent epigenetic changes in DNA methylation that are associated with disease-related phenotypes [2], [3]. This suggests that the prenatal environment (‘nurture’) can persistently influence the expression of DNA sequences (‘nature’) [4]. Recent studies stress that variation in DNA methylation is primarily influenced by genetic variation [5] and that the DNA sequence itself dictates the DNA methylation state of a locus [6].

Although there is evidence for distinct environmental and genetic influences on DNA methylation, it is not clear how both factors may interact and determine the DNA methylation levels at a particular locus. We at least are not aware of any such studies. Insight in these matters is of interest for the interpretation of epigenome-wide association studies (EWASs) [7] and studies investigating the developmental origins hypothesis [4]. We address this issue by further evaluating the interplay between environmental and genetic factors with respect to DNA methylation for selected regulatory loci within the *IGF2/H19* region.

The *IGF2/H19* imprinted region is one of the best-understood epigenetically controlled loci involving the methylation of various differentially methylated regions (DMRs). Previous studies reported that DNA methylation at the *IGF2* DMR0 is associated with genetic factors [8]–[10] and the prenatal environment, including periconceptional exposure to the Dutch Famine at the end of WW2 [11] and maternal folic acid supplementation [12].Therefore the methylation at selected loci in the *IGF2*/*H19* region in individuals exposed to prenatal famine may offer a special opportunity to evaluate the interplay between genetics and environment on DNA methylation.

**Figure 1:**
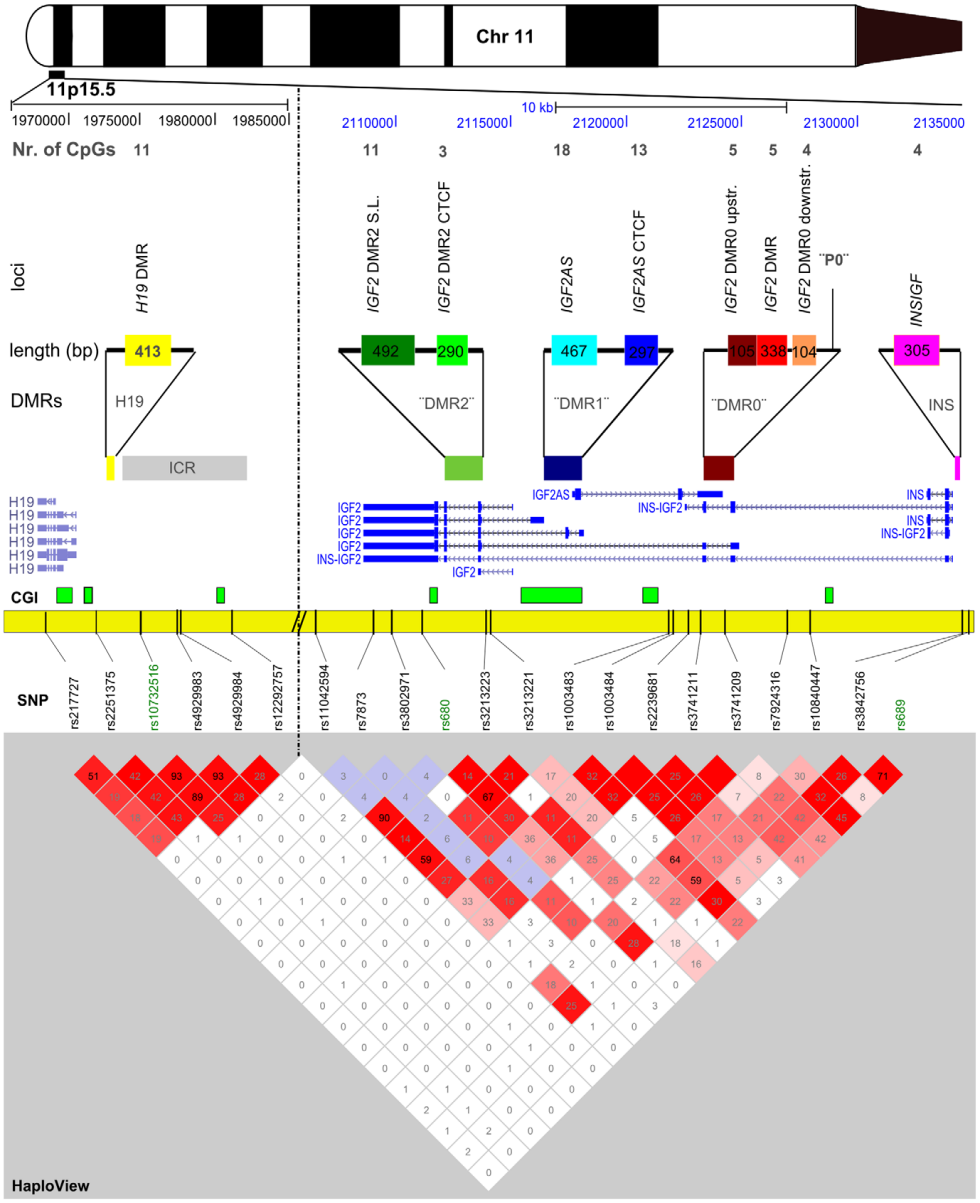
Schematic overview of the *IGF2/H19* region, measured loci and genetic variation covered. The colored boxes in the loci pane represent the DNA methylation measurements as distributed over the various functional differentially methylated regions, also defined by unique coloring (*H19* DMR, *IGF2* DMR2, DMR1, DMR0 and the *INS* promoter). The number of CpG sites measured per locus is given above the locus names. The gene structure, as defined by Refseq, is given together with the CpG islands (“CGI”, bright green). The yellow bar presents the chromosome, with the various measured SNPs marked by bars. In the HaploView pane the D′ between SNPs is given in the color scale, while the R-squared is given in numeric values in the boxes.

The correct mono-allelic expression of genes in *IGF2*/*H19* region in somatic cells is regulated by several DMRs (Figure 1) [13], [14]. Going from centromere to telomere, the first DMR is the imprinted insulin promoter (*INS*) [15], which also influences the neighboring insulin-like growth factor 2 (*IGF2*) gene [16]. *INS* forms a fusion transcript between *INS* and *IGF2* during early development, called *INSIGF* [17] and DNA methylation at this locus is correlated with *INS* transcription [18]. The next DMR is *IGF2* DMR0 (alternate name *IGF2* DMR) at which abnormal DNA methylation is associated with bi-allelic expression of *IGF2* [19], [20]. The *IGF2* DMR1, within a large CpG island overlapping the *IGF2AS* promoter (alternate name *PEG8*), is reported to have an insulator function and bind CTCF [21]. *IGF2* DMR2 was also reported to act as an insulator and to bind CTCF and aberrant DNA methylation at the locus is associated with a loss of imprinting [22]. The final DMR is located in the promoter of the *H19* transcript that directly flanks the imprinting control region. Aberrant DNA methylation at this DMR is correlated with a loss of imprinting and over-expression [23].

Here, we present an in-depth characterization of DNA methylation differences at nine regulatory loci within five DMRs across the *IGF2/H19* region between 60 individuals exposed periconceptional to the Dutch Famine and 60 same-sex sibling controls without prenatal famine exposure. All individuals are part of our ongoing Dutch Hunger Winter Families Study [24]. We examined if the famine associations are locus specific or extend to multiple functional loci. We also examined a measure of global methylation to compare the locus-specific associations with possible overall genomic effects after famine exposure. Moreover, we evaluated the association between *IGF2/H19* methylation and common genetic variation in the sibling pairs by genotyping tagging SNPs. Finally, we tested if the associations between famine exposure and genetic variation are independent and contrasted the effect sizes of these associations to describe the relative contribution of ‘nature’ and ‘nurture’ to variation in DNA methylation at *IGF2/H19*.

## Results

### Analysis of *IGF2*/*H19* methylation

Within the five DMRs, nine methylated loci were reported to regulate imprinting and expression of *INS*, *INSIGF*, *IGF2* and *H19* (Figure 1) [9], [17]–[22], [25]–[27]. We analyzed DNA methylation at one locus in the *INS* promoter (*INSIGF*), three in *IGF2* DMR0 (*IGF2* DMR0 downstr., *IGF2* DMR and *IGF2* DMR0 upstr.), two in *IGF2* DMR1 (*IGF2AS* CTCF and *IGF2AS*) and two in DMR2 (*IGF2* DMR2 CTCF and *IGF2* DMR2 S.L.) and one in the *H19* DMR (*H19* DMR). Information on the functionality of these loci is provided in the materials and methods section. The precise genomic locations are given in a .BED file (BED S1) and in table S1. Information on the individual CpG dinucleotides measured within each locus is given in table S2. We measured DNA methylation at these loci in 60 individuals with periconceptional famine exposure and 60 unexposed, same-sex siblings. DNA methylation was quantitatively assessed by mass spectrometry (Epityper [28]), which quantifies the number of methylated and unmethylated fragments following bisulfite PCR and base specific cleavage.

**Figure 2:**
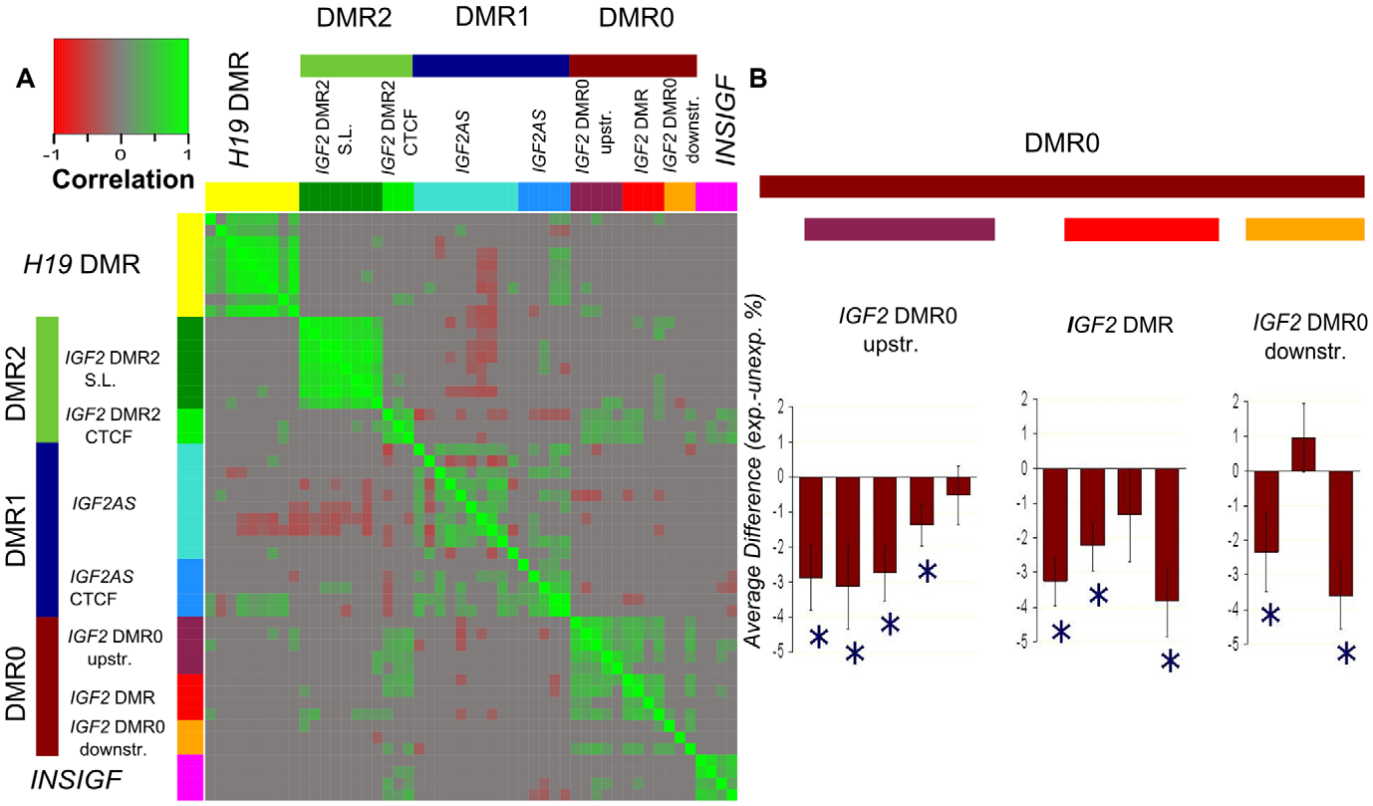
DNA methylation across multiple loci. The colored bars present DNA methylation measurements of the various loci and their grouping in the final analyses. **A**. The correlation of the DNA methylation of CpG dinucleotides within *IGF2/H19.* Each square block represents the pair wise correlation between two CpG dinucleotides in 120 individuals, the 60 individuals exposed periconceptional to famine and their same-sex siblings. Only significant correlations are shown in a color gradient from red (ρ = −1) to gray (ρ = 0 or N.S.) to green (ρ = +1). **B**. The average within pair difference in DNA methylation (%) between the famine exposed and their same-sex sibling controls for the 3 amplicons measured in the *IGF2* DMR0. A * denotes if the individual fragment containing one or multiple CpG sites is significantly different between the exposed and unexposed (P<0.05).

Inspection of DNA methylation patterns showed that DNA methylation at different loci assayed within a DMR was correlated (Figure 2A), except for DMR2. In DMR2, methylation at the *IGF2* DMR2 CTCF locus (a CTCF binding site [21]) was not correlated with the *IGF2* DMR2 S.L. locus (a DNA stem loop structure [27]). In view of the high within DMR correlation, the three loci assayed for *IGF2* DMR0 and the two for *IGF2* DMR1 were also analyzed as a single locus. Positive correlations were observed between DMRs, in particular between DMR0, DMR1, DMR2 CTCF and *INSIGF*. Interestingly these loci are located up to 10 kb apart. To a lesser extent, correlations were also observed between *H19* DMR and DMR2 S.L.

### Prenatal famine exposure and IGF2/H19 methylation

We previously reported on the association of methylation at the *INS* promoter locus *INSIGF* with periconceptional famine exposure [29]. The *INSIGF* methylation was 1.5% lower in exposed individuals as compared to the unexposed siblings (P_BH_ = 0.015 after Benjamini–Hochberg adjustment for multiple testing). Expressed relative to the standard deviation in controls of 2.6%, this difference corresponds to a standardized effect size of −0.6 SD units (Table 1).

**Table 1:** The associations between periconceptional famine exposure and DNA methylation.

Locus	Controls Methylation (sd) %^1^	Exp. –Unexp. (%)^2^	Effect size^3^	P	P_BH_ 4
*INSIGF*	*84.8 (2.6)*	*−1.5*	*−0.6*	*4.0×10^−3^*	*0.015*
*IGF2 DMR0*	*53.3(3.3)*	*−2.0*	*−0.6*	*2.7×10^−4^*	*2.9×10^−3^*
*IGF2* DMR0 downstr.	71.2(3.3)	−1.6	−0.6	0.024	0.038
*IGF2* DMR	51.5(5.5)	−2.4	−0.5	5.3×10^−4^	2.9×10^−3^
*IGF2* DMR0 upstr.	44.2(4.0)	−1.9	−0.4	6.0×10^−3^	0.017
*IGF2 DMR1*	*6.4(0.8)*	*0.4*	*+0.5*	*9.0×10^−3^*	*0.020*
*IGF2AS* CTCF	4.3(0.9)	0.3	+0.3	0.040	0.049
*IGF2AS*	8.6(1.0)	0.4	+0.4	0.038	0.049
*IGF2 DMR2*					
*IGF2* DMR2 S.L.	49.8(6.3)	0.4	+0.1	0.78	0.78
*IGF2* DMR2 CTCF	50.8(2.7)	−1.2	−0.4	0.012	0.022
*H19 DMR*	*30.6 (2.6)*	*−0.5*	*−0.2*	*0.36*	*0.39*

1 The average DNA methylation in the unexposed sibling controls and the standard deviation of this average, both given in %.

2 The within pair difference in DNA methylation resulting from a linear mixed model corrected for age at blood drawl, correlations between CpG sites, bisulfite conversion batch and with a random effect for sib ship and a random slope for exposure status.

3 The effect size of the within pair difference in relation to the standard deviation in the population.

4 Two-sided P-value, Benjamini-Hochberg (‘FDR’) corrected for 11 tests.

*IGF2* DMR0 methylation was lower in the exposed siblings (Δ = −2.0%; P_BH_ = 2.9×10^−3^), corresponding to a standardized effect size of −0.6 SD units, similar to what was also observed for *INSIGF* (table 1). When analyzed separately, all 3 loci measured within the *IGF2* DMR0 (a locus previously analyzed in this study population [11] and two newly measured loci flanking that locus), were similarly associated with prenatal famine exposure (figure 2B and Table 1). Subsequent analysis of individual CpG dinucleotides in these loci showed a significant association for nine out of twelve CpG containing fragments (table S2, Figure 2B).

Methylation at the *IGF2* DMR1 was higher in exposed individuals as compared with controls (P_BH_ = 0.020), but the absolute difference in DNA methylation was very small (Δ = +0.5%). The difference corresponds to a standardized effect size of 0.5 SD units, similar to that observed for *INSIGF* and *IGF2* DMR0, which is related to the lower inter-individual variation at DMR1 (Table 1). DNA methylation at the two individual loci measured within *IGF2* DMR1 was likewise modestly higher in those exposed periconceptional (P_BH_ = 0.049). In contrast to other associated DMRs, only a small minority of CpG dinucleotides within the two DMR1 loci (3/24) were statistically significant (table S2).

DNA methylation of the two loci measured in *IGF2* DMR2 was not correlated and therefore analyzed separately. The *IGF2* DMR2 CTCF locus showed a significant association with famine exposure (Δ = −1.2%, P_BH_ = 0.02). With an effect size of −0.4 SD units this association was similar to those found for the other investigated *IGF2* DMRs (Table 1). All three individual CpG sites showed a lower methylation level in the exposed compared to the controls and for two out of three the difference was statistically significant (table S2). The *IGF2* DMR2 S.L. locus was not associated with famine exposure (P_BH_ = 0.78) and DNA methylation at the *H19* DMR was also not significantly associated with prenatal famine exposure (P_BH_ = 0.39).

To evaluate whether the generally lower DNA methylation at *IGF2* DMRs was related to an overall lower genomic DNA methylation, we measured LINE-1 methylation, an estimate of global methylation [30]. LINE-1 methylation was 61.2% (SD 1.4%) in controls and this was not different from individuals with periconceptional famine exposure (Δ = −0.4%, P = 0.15, table S2). This result confirms our previous report that prenatal famine had no effect on three other measures of global methylation in this study population [31] and indicates the absence of a general trend towards either reduced or increased genomic DNA methylation.

### Genetic variation and IGF2/H19 methylation

To capture common genetic variation at the *IGF2*/*H19* locus, 21 SNPs were genotyped. The SNPs were selected as tagging SNPs from the HAPMAP CEU panel or selected from literature (figure 1, table S3 and S4). Linkage disequilibrium (LD) analysis indicated that 16 of the 21 SNPs captured the common genetic variation marked by these SNPs (R^2^>0.9). Of these sixteen SNPs, four were located in the *H19* region and twelve in the *IGF2*-*INS* region; no LD was observed between the two regions (Figure 1). Genotype frequencies were similar in exposed individuals and unexposed siblings (P>0.13, without multiple testing correction).

**Figure 3:**
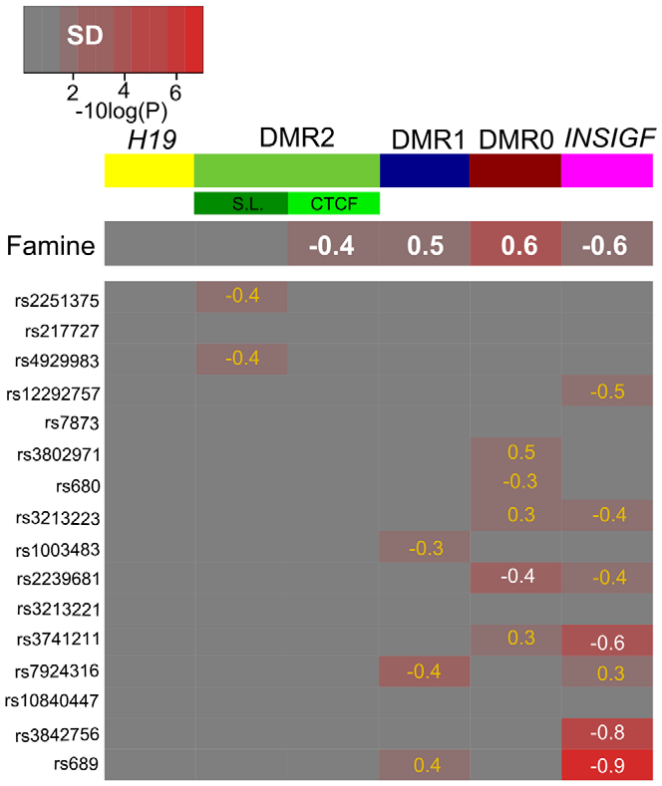
The association of prenatal famine and genetic variation *in cis* with *IGF2*/*H19* methylation. The associations between famine or genotype with DNA methylation. The p-value of the association (−log10 scale) is given in a color scale from non significant (gray) to highly significant (bright red). DMRs are denoted by the colored bars in top of the figure. The effect sizes are given in standardized SD-scores. In a white font are the associations significant after multiple testing correction. The nominally significant associations are denoted in orange.

We then explored which of these sixteen tagging SNPs was associated with DNA methylation at the *IGF2*/*H19* DMRs in the sixty sib ships (N = 120). DNA methylation at *INSIGF* was significantly associated with SNPs in *IGF2* (rs3741211 [β = −1.5% per minor allele, P_BH_ = 3.4×10^−3^]) and *INSIGF* (rs3842756 [β = −2.0%, P_BH_ = 3.9×10^−4^] and rs689 [β = −2.3%, P_BH_ = 7.1×10^−6^]) (Table 2). The standardized effect size of associations increased with decreasing distance from the DMRs (from −0.6 to −0.9 SD units per minor allele, Figure 3). The largest effect size was observed for the association of rs689, which is in perfect LD with the *INS* VNTR I/III alleles in Caucasian populations [32], with *INSIGF* methylation (−0.9 SD; P_BH_ = 7.1×10^−6^). Other nominally significant associations with *INSIGF* (P<0.05 and P_BH_>0.05) are reported in table S7 and in figure 3.

**Table 2:** The significant associations between SNPs and DNA methylation.

Locus	Meth. (sd) in %	Effect of genotype on DNA methylation^3^				
*INSIGF*	84.8(2.6)	SNP	*B* 1	effect size^2^	P	P_BH_ 4
		rs3741211	−1.5	−0.6	1.1×10^−4^	3.4×10^−3^
		rs3842756	−2.0	−0.8	8.2×10^−6^	3.9×10^−4^
		rs689	−2.3	−0.9	7.4×10^−8^	7.1×10^−6^

*IGF2* DMR0	53.3(3.3)	SNP	*B*	effect size	P	P_BH_
		rs2239681	−1.3	−0.4	1.1×10^−3^	0.027

1 The change in average DNA methylation in % with each minor allele. From a linear mixed model corrected for age at blood drawl, correlations between CpG sites, bisulfite conversion batch and with a random effect for sib ship and a random slope for exposure status. The genotype was added as continues variable.

2 The effect size of the beta in relation to the variation in DNA methylation in the population.

3 The associations that survive multiple testing correction; a complete overview off all the results is given in tables S5A–C.

4 Two-sided P-value, after Benjamini-Hochberg correction.

For *IGF2* DMR0, rs2239681 was associated with DNA methylation (β = −1.3%, P_BH_ = 0.027; Figure 3, table S7). For two SNPs nominally significantly associations were observed, which included rs680 (i.e. “*Apa*I”, β = −1.1, P = 0.017 [P_BH_ = 0.17]), for which we reported an association previously [8].

For *IGF2* DMR1 no statistically significant associations were observed after accounting for multiple testing. One of the three nominally significant associations was rs689 marking the *INS* VNTR located near *INSIGF* (Figure 3, Table S6).

The two loci in *IGF2* DMR2 (CTCF and S.L.) were analyzed separately in contrast to the loci comprising the other DMRs because the methylation levels were not correlated (Figure 2A). No associations were observed for *IGF2* DMR2 CTCF and S.L. when accounting for multiple testing. Two nominally significant association were found for the *H19* SNPs rs2251375 and rs4929983 (tagging rs10732516 in the ICR's sixth CTCF binding site [33]) and *IGF2* DMR2 S.L. DNA methylation (Figure 3, Table S5, Table S6). For *H19* DMR, no associations were observed in line with earlier observations [8].

### Prenatal famine exposure and genetic variation

Expressed as standardized effect sizes, the average DNA methylation difference between exposed individuals and unexposed siblings was 0.5 SD units for significantly associated DMRs (P_BH_<0.05). A similar average of 0.5 SD per minor allele was observed for associated SNPs (P_BH_<0.05; Figure 3). Since the methylation at several loci was associated with both famine exposure and SNPs in these sibling pairs, the analyses for associations between prenatal famine and DMR methylation were repeated with adjustment for the SNPs significantly associated with DMR methylation (either nominally or after correction for multiple testing). All famine associations remained statistically significant and the effect sizes remained similar (table S8). Similarly, the genetic associations were not affected after adjustment for prenatal famine exposure (table S9).

Next, we tested for possible interactions between famine exposure and genetic variation with respect to the DNA methylation levels at *IGF2*/*H19*. No interaction was significant after control for multiple testing. Of all tested interactions, only the interactions between prenatal famine exposure and *INSIGF* SNPs rs3842756 (P = 0.048) and rs689 (P = 0.016) in relation to *IGF2* DMR1 methylation were nominally significant. The effect of prenatal famine exposure and genetic variation on DNA methylation at the DMRs therefore appear to be additive.

## Discussion

We studied the relations between periconceptional famine exposure, genetic variation and DNA methylation at DMRs in the imprinted *IGF2/H19* region. Famine exposure was associated with widespread but modest differences in DNA methylation across multiple DMRs within the *INS* and *IGF2* transcribed region. Associations of SNPs with DNA methylation at the *IGF2*/*H19* DMRs were likewise common and modest. When expressed relative to the variation in DNA methylation in the population, prenatal famine and SNPs were associated with similar effect sizes, around 0.5 SD units. Periconceptional famine exposure and genetic variation were associated with DNA methylation at the same DMRs and these associations were independent of each other.

We previously reported a decrease in *IGF2* DMR0 methylation after periconceptional exposure to famine [11]. DNA methylation at two loci directly flanking this locus was similarly associated, extending the affected region in DMR0 to a region of 1.5 kb. Significant differences in DNA methylation were also observed for *IGF2* DMR1, DMR2 and *INSIGF*. Despite being widespread and statistically significant, the absolute differences between the exposed and their siblings varied and were modest (<3.6 percent points) and represent a moderate change when set out against the inter-individual variation (∼0.5 SD units). Long-term functional consequences of such small absolute changes, which were also reported for other exposures [12], [34]–[36], remain to be established.

Wide-spread small changes were suggested to be a plausible mechanism by which epigenetic fine-tuning of pathways may occur [37]. In this respect it is of interest to note that the magnitude of the effect sizes was similar for all DMRs. We are the first to show that a prenatal environmental exposure may influence DNA methylation at multiple distinct regulatory sites within the same gene. Small variations in DNA methylation at particular loci associated with prenatal human environmental exposures [12], [29], [34], [35], [38], risk factors for disease [39], [40], or clinical end-points [41], may represent variation at multiple sites.

The similarity of the effect sizes among the DMRs may also reflect a molecular remnant of differences in gene expression during the periconceptional period among famine exposed individuals. A study in mouse oocytes showed that active transcription influences the DNA methylation deposition at *Gnas* and other imprinted DMRs [42]. In addition, changes in gene expression during late mouse liver development also influences DNA methylation at various genes [43]. In view of these studies, the decrease in DMR0 and DMR2 methylation may reflect a temporary decrease in *IGF2* transcription. These differences may initially have been larger than currently observed, as they were measured six decades after the actual exposure. After the exposure, the differences may have been diluted by other environmental influences [44] and stochastic changes accumulating during ageing [45]. However, the subtle nature of the DNA methylation differences on a population level may also be inherent to the noise in the epigenetic response to environmental exposures [46].

Another aspect potentially contributing noise is the cellular heterogeneity of whole blood, the sample type currently studied. However, *IGF2* DMR0 methylation was shown not to be influenced by cellular heterogeneity, nor was there a difference between buccal cells and blood for this DMR [45]. Although this does not exclude differences between blood cell types for loci within *IGF2*/*H19*, including DMR0 [10], this implies that the combination of the small size of these differences between cell types in combination with the limited variation in proportions of these cell types in blood between individuals is unlikely to have influenced our observations for this imprinted locus. Moreover, animal studies showed that both an exposures during gametogenesis [3] and the early post conception stage [47] can both lead to epigenetic differences observed in multiple tissues in adults, presumably because they were propagated soma-wide. Therefore, if differences were induced early in human development, for example by periconceptional famine exposure, they could likewise be propagated soma-wide and be present across cell-types and tissues [48]. We currently do not have access to other (internal) tissues in our cohort to test this hypothesis, an important issue in epigenetic epidemiology [49].

Previous studies suggest that 95% of reported associations between SNPs and DNA methylation occurred for SNPs located within 149 kb of the CpG dinucleotides [50] with a peak at a physical distance of just 45 bp [51]. Indeed, associations were most frequent between DMRs and adjacent SNPs in our study. The effect sizes we found are smaller than those reported in some genome-wide studies [52], but comparable to those reported by Bell *et al*. for SNPs influencing DNA methylation at *FTO* [53]. Our study is comparable in size to these studies. We confirm an association for SNPs and *IGF2* DMR methylation as seen in a study among mono- and dizygotic twins [8] and our findings are also in line with results from two larger studies in unrelated individuals and twins for *IGF2* DMR and *H19* [33], [54].

The most significant association between genetic variation and *IGF2*/*H19* DNA methylation was observed for rs689 and *INS* promoter methylation (*INSIGF*) (effect size of −0.9 SD units, or −2.3% percent points per minor allele, ∼140 bp distance). SNP rs689 is a perfect proxy for the class I and III alleles of the *INS* VNTR in European populations [32] and associations between the *INS* VNTR and type 2 diabetes (T2D), metabolic syndrome and early growth have been frequently reported by some [55], [56], but also refuted by others [56]–[58]. The *INS* VNTR was reported to influence insulin gene expression [59] and DNA methylation at the *INS* promoter were found to be correlated with *INS* expression, HbA_1c_ levels and T2D [18]. Our findings that the *INS* VNTR is associated with *INS* promoter methylation may shed new light on the association of the VNTR with *INS* expression and the metabolic syndrome [60]. This assumes of course that our findings in blood cells extend to relevant tissues directly involved in these conditions. Tissue specificity is not only an issue for associations between the (prenatal) environment and DNA methylation [49], but also for SNP-DNA methylation associations: a sizeable number of associations between SNPs and DNA methylation in a large study on DNA methylation from four different brain regions were found to be tissue specific [51].

In conclusion, our observations that *INSIGF* and *IGF2* DMR0 DNA methylation levels are independently associated with genetic and early environmental factors is relevant for the design and interpretation of epigenetic association studies involving *IGF2*/*H19*. Our analyses indicate that Mendelian randomization approaches are feasible to infer causality for associations observed between DNA methylation and disease phenotypes [61]. Secondly, our results will be relevant for the interpretation of epigenome-wide association studies as genomic and environmental forces may act in tandem through the epigenome on the phenotype of interest. There may be similar and additive effects of ‘nurture’ and ‘nature’ on DNA methylation within *IGF2*/*H19*. Thus, for some loci, epigenetics may be the information layer in which the classical contrast between ‘nurture’ and ‘nature’ debate comes to a modern molecular synthesis [62].

## Materials and Methods

### Study population

The characteristics and detailed recruitment of the Hunger Winter Families Study were described previously [24]. In short, study subjects were selected from births between 1943–1947 at three institutions in famine-exposed cities (the midwifery training schools in Amsterdam and Rotterdam and the Leiden University Medical Center) and include singleton births exposed to famine *in utero*, same-sex sibling controls not exposed during *in utero* development and time controls conceived and born either just before or just after the famine. Ethical approval for the study was obtained from the participating institutions and all participants provided written informed consent.

Despite the war, nutrition in the Netherlands had generally been adequate until October 1944 [63]. Thereafter, supplies became increasingly scarce. By the end of November, the level of official supplementary rations, which eventually consisted of little more than bread and potatoes, had fallen below 1,000 kcal per day, and by April 1945 they were as low as 500 kcal per day [64]. Since the Dutch population was well fed before and after the famine and since the famine period was shorter than the nine months of human gestation, individuals can be defined by exposure during specific periods of their development in uterus.

In this study we use a subset of the 313 singleton births in the larger cohort [24] who were exposed to the Dutch famine *in utero* and who completed a clinical examination together with a same-sex sibling without prenatal famine exposure. Whole blood was collected from all individuals for DNA extraction during this examination. We focused on individuals whose mother was exposed to famine around the moment of conception and in the first 10 weeks of gestation. These ‘periconceptionally’ exposed individuals were defined as births with a mother's estimated last menstrual period between November 28, 1944 and May 15, 1945. This group includes 60 individuals of whom 28 are male and 32 are female (age at examination and blood draw 58.1 y [SD, 0.35 y]). As controls we used their unexposed same-sex sibling for (partial) genetic and gender matching (age at examination and blood draw 57.0 y [SD, 5.9 y]). 24 controls were conceived and born before the famine (11 male, 13 female) and 36 individuals were conceived and born after the famine (17 male and 19 female). The studied population includes 120 individuals in total.

### DNA methylation assay design

We used BLAT against genome build 36 in the UCSC genome browser [65] to find the locations in 11p15.5 mentioned in the various original articles [9], [17]–[22], [25]–[27]. We provide a .BED file showing the various locations assayed and the location of the elements from the original articles on which they were based with the (BED S1). From the centromere outwards the loci were chosen as follows (Figure 1).

The *INSIGF* locus was previously measured by us [29], [45], [66], is imprinted [17] and DNA methylation is correlated with expression [18]. Three loci were designed for the *IGF2* promoter region, *IGF2* DMR0, at which hypomethylation is associated with bi-allelic expression [19], [20]. *IGF2* DMR upstream (upstr.) and *IGF2* DMR downstream (downstr.) directly flank the *IGF2* DMR locus that we measured previously in this cohort [11]. *IGF2* DMR downstr. is located next to the “P0” promoter, which interacts with the imprinting control region [26]. Two loci were designed to cover the promoter region of the *IGF2*AS transcript, which we name *IGF2* DMR1. One of these loci shows CTCF binding activity (*IGF2*AS CTCF) and one locus demonstrated insulator activity (*IGF2*AS) [21]. Two loci overlap the *IGF2* DMR2, of which aberrant DNA methylation has been linked to loss of imprinting (LOI) [22] and male fertility [25]. *IGF2* DMR2 CTCF overlaps a CTCF binding site [21], while IGF2 DMR2 S.L. overlaps a highly conserved DNA stem loop structure [27]. Last, the *H19* DMR locus was previously designed [8] to measure part of the *H19* promoter at which aberrant DNA methylation was found to correlate with LOI and over expression [23]. Several primer pairs for the sixth CTCF binding site in the ICR from literature and from our own design were tested, but gave a-specific PCR products or amplification of genomic, non-bisulfite treated DNA in our automated work-flow. We also estimated global methylation using an assay for LINES-1 [30], based on the same technique.

Primers were designed using Methprimer [67]. The resulting primer and amplicon locations were checked against the latest version of dbSNP and for their spectrum characteristics with the R package RSeqMeth [68]. The sequences of the primers used in our study and the genomic locations they amplify are given in table S1.

### DNA methylation measurements

Genomic DNA from whole blood was isolated using the salting-out method. Bisulfite treatment on 500 ng of genomic was performed with the EZ 96-DNA methylation kit (Zymo Research) with overnight bisulfite incubation according to the supplier's protocol. The 60 sibling pairs were randomly distributed over two 96 well plates with similar proportions of male and female pairs on each plate. DNA methylation was quantitatively assessed for each locus using the mass spectrometry based Epityper assay (Sequenom, USA) in triplicate using the manufacturers' protocol on one 384 well plate. PCR was performed with the following cycling protocol: 15 minutes at 95°C, four rounds of 20 seconds at 95°C, 30 seconds at 65°C, 1 minute at 72°C; followed by forty rounds, 20 seconds at 95°C, 30 seconds at 58°C and 1 minute at 72°C; ending with 3 minutes at 72°C. Processing of the Epityper data has been described in detail previously [11], [29], [45], [66]. In short only measurements for CpG dinucleotides containing fragments for which 2 out of 3 measurements were successful, the standard deviation (SD) was smaller than 10% and for which the overall measurement success rate in the population was higher than 75% were included in the final analyses. Before data filtering the SD between the triplicate measurements ranged from 2% to 5.4%, after data filtering this measure ranged from 1.5% to 3.5%. We used the average of these triplicate measurements for the analyses. For each measurement we incorporated non-bisulfite converted genomic DNA and negative controls to check for a-specific amplification and PCR artifacts. None were found. Bisulfite conversion was assessed using the MassArray R package [69], which uses fragments containing a TpG and a cytosine to assess the conversion. No indication for an incomplete bisulfite conversion or PCR amplification of non-bisulfite converted DNA was observed.

### SNP selection and genotyping

From the combined HapMap phase I, II and III data [70] the CEU genotype data were downloaded for the region of the Refseq *H19* and the *IGF2* and *INSIGF* transcripts with an additional 150 kb at both the 5′ and 3′ ends. These data were visualized in Haploview [71] for both regions separately. Based on the linkage disequilibrium (LD) structure one or more HaploView defined LD blocks were selected, covering the entire region for which DNA methylation was measured. For *INS* and *IGF2* this resulted in a region stretching from rs11042594 to rs3842748 (NCBI 36 chr11:2,073,729-2,137,971) and for *H19* in a region stretching from rs3741219 till rs3890907 (NCBI36 chr11:1,973,195-1,984,719). In addition, we selected 16 SNPs in these regions that have been associated with relevant phenotypes such as being born small for gestational age [54], [72], birth weight [73]–[75], body mass index [55], [56], [60], [76], [77], type two diabetes [60], postnatal growth [75] and *IGF2* levels [74], [78] or with DNA methylation at *IGF2* DMR or *H19* DMR [8]. Twelve of these SNPs were also in the CEU HapMap set. A complete overview is given in tables S3 and S4. We used HaploView pairwise tagging (r^2^>0.8) and used force include on the candidate SNPs if they were part of HapMap to obtain a set of tagging SNPs for the region. We used only SNPs with a minor allele frequency higher than 0.1 because of the limited sample size of our cohort. For *H19* rs10732516 and for *INSIGF* rs680, rs3213223 and rs1003484 were added to this list since they are not part of the CEU HapMap set. We thus obtained 10 SNPs for *H19* and 23 SNPs for *IGF2* and *INSIGF*. Genotyping was performed using Sequenom MassARRAY iPLEXGold with the exception of rs10732516. This latter SNP was measured using an ABI 3710 because of the highly repetitive nature of this region. The forward and reverse primers were as follows for this assay: Forward 5′- ACG TTT CCA CGG GCG A -3′, Reverse 5′-GCC CTA GTG TGA AAC CCT TCT-3′. This amplifies hg18 region chr11:1977715-1977936. Amplification was performed with the following conditions: 15 minutes at 95°C, thirty-five times 30 seconds 94°C, 60 seconds 55°C, 30 seconds 72°C with a final step of 3 minutes at 72°C.

The complete list of SNPs, their biological significance, success rate, the minor allele frequency and the test for Hardy-Weinberg equilibrium is given in table S3 and S4. In short, for four *H19* and three *INSIGF* SNPs no iPLEX probe design was possible due to the close proximity of other SNPs. Three SNPs were not polymorphic in this Dutch population, one SNP had a lower than 95% success rate, and two SNPs were out of Hardy-Weinberg (P<0.002) according to HaploView and were thus discarded from the analyses. Since not meeting the Hardy-Weinberg criterion can be a sign of selection we tested these two SNPs (rs4320932 and rs4341514) for frequency differences between the exposed and unexposed, but found none (P>0.2). After checking the LD structure in this population of the successfully measured SNPs (figure 1), several SNP were found to be in very high pair-wise LD (R^2^>0.9), allowing us to restrict the number of SNPs to test. This resulted in a final set of four *H19* and twelve SNPs in *IGF2* and *INS* that captured the common genetic variation at *IGF2/H19.* The sixteen SNPs either occurred in CpG dinucleotides themselves (‘CG SNPs’, Table S10) or were in LD with such SNPs limiting the possibilities to contrast CG SNPs with non-CG SNPs.

### Statistics

We tested for within-pair differences in DNA methylation between exposed individuals and their non-exposed same-sex sibling by applying linear mixed models. With these models the correlation between adjacent CpG sites can be taken into account and all available raw but incomplete data can be used for modeling and control for possible confounders. For an amplicon the difference in DNA methylation between siblings was tested by entering as fixed effects the exposure status (exposed vs. unexposed), a unique identifier for each CpG site within the locus, age at blood draw and the bisulfite batch. To specify a within-sib-pair design, a family (pair) identifier was included as a random effect with intercept. To model the correlation in DNA methylation within an individual we make use of the fact that each family consists of an exposed and same-sex sibling control, therefore adding an exposure status to the random effect as a random slope. This in effect functions as if the individual identifier was added as an additional random effect in our design. This model option allows us to use the same model for both multiple CpG sites and single CpG sites, allowing a unified statistical analysis on all data. The REML likelihood method was used for the model fitting. The difference in DNA methylation for individual CpG sites was calculated with the same model but without the identifier for CpG site. This single CpG linear mixed model yields the same outcome as a paired t-test if no data are missing and no correction for covariates such as age are applied. Some DMRs show an association with age [11], since half of the pairs have a sibling born before the war and half after we could correct for this possible confounder.

The average DNA methylation level of loci was computed using imputed estimates for missing values, since calculating the average with missing values can skew the average and estimate of the normal variation in the population because of the sometimes large differences in DNA methylation levels between different CpG dinucleotides within a locus. Imputed values were retrieved from the same linear mixed models, which can estimate methylation of CpG sites if data is missing, using information of other CpG sites and the covariates (bisulfite batch, sex and age). The imputed values were never used for any of the analyses. We did not observe significant differences in variance in DNA methylation between the exposed and unexposed (Levene's test for homogeneity P>0.05).

To test for associations between DNA methylation and a genotype, the model was extended with the genotype as a fixed effect with the genotype coded as 0 (for common allele homozygous), 1 (heterozygous) or 2 (homozygous for the rare allele) and added as a continuous variable. Finally, to test for interactions between famine exposure and genetic variation an interaction term was added to the model as a fixed effect. In all cases the main effects were also included in the model. We also tested for an interaction between prenatal famine exposure and sex on DNA methylation of all DMRs, but no significant interactions were found, except for *INSIGF* as previously reported [29].

Differences in genotype frequency between the exposed individuals and their unexposed siblings were evaluated using Chi-square test. All analyses were performed in SPSS version 17.0. To visualize correlations in DNA methylation between individual CpG containing fragments and the significance of associations, heatmaps were generated in R version 2.12.1 using the “heatmap.2” function of the gplots package. Multiple testing correction was performed according to the method developed by Bejamini and Hochberg, better known as ‘FDR’ (false discovery rate) correction using the R base ‘p.adjust’ function.

## Supporting Information

BED S1 **A .BED file for the UCSC genome browser with all the locations in the** ***IGF2*** **/** ***H19*** **locus investigated.**
(BED)

Table S1 **The primers and amplified regions.** 1. The sequence of the forward primer, for Epityper a tag with the following sequence is added 5′: AGGAAGAGAG 2. The sequence of the reverse primer, for Epityper a tag with the following sequence is added 5′: CAGTAATACGACTCACTATAGGGAGAAGGCT PCR was performed with the following cycling protocol: 15 minutes at 95°C, 4 rounds of 20 seconds at 95°C, 30 seconds at 65°C, 1 minute at 72°C; followed by 40 rounds, 20 seconds at 95°C, 30 seconds at 58°C and 1 minute at 72°C; ending with 3 minutes at 72°C.
(DOC)

Table S2 **Information on individual CpG containing fragments.** 1. CpG containing fragments (e.g. ‘CpG units’): excluded were fragments containing possible SNPs in CEU (by HAPMAP or 1000genomes), a measurement success rate below <75% or (partial) overlap with other units. 2. Mean methylation in %, based on the raw data. 3. the variation (in %) in the controls 4. The average within pair difference from a Linear Mixed Model, corrected for age and bisulfite batch. 5. The P value belonging to the within pair difference.
(DOC)

Table S3 **The genotyping results for the H19 LD block.** 1. Several SNPs were chosen from the HAPMAP CEU panel as tagging SNPs for the region, also several candidate SNPs were added. Some were both candidate as HAPMAP tagging SNPs. 2. Success rate of the genotyping. 3. Several SNPs could not be measured, one SNP had a low success rate and two SNPs were in perfect LD (r^2^>0.9) with another SNP in these individuals and thus not included in the final analysis. 4. The P value resulting from a test for Hardy-Weinberg disequilibrium, significant threshold is P<0.002 because of multiple testing.
(DOC)

Table S4 **The genotyping results for the INSIGF LD blocks.** 1. Several SNPs were chosen from the HAPMAP CEU panel as tagging SNPs for the region, also several candidate SNPs were added. Some were both candidate as HAPMAP tagging SNPs. 2. Success rate of the genotyping. 3. Several SNPs could not be measured, one SNP had a low success rate and two SNPs were in perfect LD (r^2^>0.9) with another SNP in these individuals and thus not included in the final analysis. 4. The P value resulting from a test for Hardy-Weinberg disequilibrium, significant threshold is P<0.002 because of multiple testing.
(DOC)

Table S5 **The effect of the tagging SNPs on DNA methylation.** The effect of the rare allele on DNA methylation, assuming an additive model. The beta is the change in average DNA methylation (%). For instance, a beta of 1.0 means that each allele increases the amount of DNA methylation at that DMR with 1.0%. The P value is given followed by the P value corrected for multiple testing (FDR).
(DOC)

Table S6 **The effect of the tagging SNPs on DNA methylation.** The effect of the rare allele on DNA methylation, assuming an additive model. The beta is the change in average DNA methylation (%). For instance, a beta of 1.0 means that each allele increases the amount of DNA methylation at that DMR with 1.0%. The P value is given followed by the P value corrected for multiple testing (FDR).
(DOC)

Table S7 **The effect of the tagging SNPs on DNA methylation.** The effect of the rare allele on DNA methylation, assuming an additive model. The beta is the change in average DNA methylation (%). For instance, a beta of 1.0 means that each allele increases the amount of DNA methylation at that DMR with 1.0%. The P value is given followed by the P value corrected for multiple testing (FDR).
(DOC)

Table S8 **Famine associations corrected for significant SNPs.** For each locus the beta for the association with famine is given, now corrected for the SNPs showing (nominally) significant associations with this locus, followed by the corrected P-value.
(DOC)

Table S9 **SNP associations with and without famine exposure correction.** The first column denotes the SNP- DNA methylation locus under investigation. Column two and three contain the beta and resulting P-value of the SNP-DNA methylation association corrected for famine exposure, the fourth and fifth the beta and P-value without famine correction. There is no notable difference between the two models.
(DOC)

Table S10 **Nine out of sixteen tagging SNPs were CpG altering polymorphisms.**
(DOC)

## References

[1] Jaenisch R, Bird A (2003). Epigenetic regulation of gene expression: how the genome integrates intrinsic and environmental signals.. Nat Genet.

[2] Bogdarina I, Welham S, King PJ, Burns SP, Clark AJ (2007). Epigenetic modification of the renin-angiotensin system in the fetal programming of hypertension.. Circ Res.

[3] Carone BR, Fauquier L, Habib N, Shea JM, Hart CE (2010). Paternally induced transgenerational environmental reprogramming of metabolic gene expression in mammals.. Cell.

[4] Waterland RA, Michels KB (2007). Epigenetic epidemiology of the developmental origins hypothesis.. Annu Rev Nutr.

[5] Gertz J, Varley KE, Reddy TE, Bowling KM, Pauli F (2011). Analysis of DNA methylation in a three-generation family reveals widespread genetic influence on epigenetic regulation.. PLoS Genet.

[6] Lienert F, Wirbelauer C, Som I, Dean A, Mohn F (2011). Identification of genetic elements that autonomously determine DNA methylation states.. Nat Genet.

[7] Rakyan VK, Down TA, Balding DJ, Beck S (2011). Epigenome-wide association studies for common human diseases.. Nat Rev Genet.

[8] Heijmans BT, Kremer D, Tobi EW, Boomsma DI, Slagboom PE (2007). Heritable rather than age-related environmental and stochastic factors dominate variation in DNA methylation of the human IGF2/H19 locus.. Hum Mol Genet.

[9] Murrell A, Heeson S, Cooper WN, Douglas E, Apostolidou S (2004). An association between variants in the IGF2 gene and Beckwith-Wiedemann syndrome: interaction between genotype and epigenotype.. Hum Mol Genet.

[10] Ollikainen M, Smith KR, Joo EJ, Ng HK, Andronikos R (2010). DNA methylation analysis of multiple tissues from newborn twins reveals both genetic and intrauterine components to variation in the human neonatal epigenome.. Hum Mol Genet.

[11] Heijmans BT, Tobi EW, Stein AD, Putter H, Blauw GJ (2008). Persistent epigenetic differences associated with prenatal exposure to famine in humans.. Proc Natl Acad Sci U S A.

[12] Steegers-Theunissen RP, Obermann-Borst SA, Kremer D, Lindemans J, Siebel C (2009). Periconceptional maternal folic acid use of 400 microg per day is related to increased methylation of the IGF2 gene in the very young child.. PLoS One.

[13] Edwards CA, Ferguson-Smith AC (2007). Mechanisms regulating imprinted genes in clusters.. Curr Opin Cell Biol.

[14] Ideraabdullah FY, Vigneau S, Bartolomei MS (2008). Genomic imprinting mechanisms in mammals.. Mutat Res.

[15] Moore GE, Abu-Amero SN, Bell G, Wakeling EL, Kingsnorth A (2001). Evidence that insulin is imprinted in the human yolk sac.. Diabetes.

[16] Ferguson LA, Docherty HM, MacKenzie AE, Docherty K (2009). An engineered zinc finger protein reveals a role for the insulin VNTR in the regulation of the insulin and adjacent IGF2 genes.. FEBS Lett.

[17] Monk D, Sanches R, Arnaud P, Apostolidou S, Hills FA (2006). Imprinting of IGF2 P0 transcript and novel alternatively spliced INS-IGF2 isoforms show differences between mouse and human.. Hum Mol Genet.

[18] Yang BT, Dayeh TA, Kirkpatrick CL, Taneera J, Kumar R (2011). Insulin promoter DNA methylation correlates negatively with insulin gene expression and positively with HbA(1c) levels in human pancreatic islets.. Diabetologia.

[19] Cui H, Onyango P, Brandenburg S, Wu Y, Hsieh CL (2002). Loss of imprinting in colorectal cancer linked to hypomethylation of H19 and IGF2.. Cancer Res.

[20] Cui H, Cruz-Correa M, Giardiello FM, Hutcheon DF, Kafonek DR (2003). Loss of IGF2 imprinting: a potential marker of colorectal cancer risk.. Science.

[21] Du M, Beatty LG, Zhou W, Lew J, Schoenherr C (2003). Insulator and silencer sequences in the imprinted region of human chromosome 11p15.5.. Hum Mol Genet.

[22] Dejeux E, Olaso R, Dousset B, Audebourg A, Gut IG (2009). Hypermethylation of the IGF2 differentially methylated region 2 is a specific event in insulinomas leading to loss-of-imprinting and overexpression.. Endocr Relat Cancer.

[23] Takai D, Gonzales FA, Tsai YC, Thayer MJ, Jones PA (2001). Large scale mapping of methylcytosines in CTCF-binding sites in the human H19 promoter and aberrant hypomethylation in human bladder cancer.. Hum Mol Genet.

[24] Lumey LH, Stein AD, Kahn HS, van der Pal-de Bruin KM, Blauw GJ (2007). Cohort profile: the Dutch Hunger Winter families study.. Int J Epidemiol.

[25] Boissonnas CC, Abdalaoui HE, Haelewyn V, Fauque P, Dupont JM (2010). Specific epigenetic alterations of IGF2-H19 locus in spermatozoa from infertile men.. Eur J Hum Genet.

[26] Nativio R, Wendt KS, Ito Y, Huddleston JE, Uribe-Lewis S (2009). Cohesin is required for higher-order chromatin conformation at the imprinted IGF2-H19 locus.. PLoS Genet.

[27] Otte K, Choudhury D, Charalambous M, Engstrom W, Rozell B (1998). A conserved structural element in horse and mouse IGF2 genes binds a methylation sensitive factor.. Nucleic Acids Res.

[28] Ehrich M, Nelson MR, Stanssens P, Zabeau M, Liloglou T (2005). Quantitative high-throughput analysis of DNA methylation patterns by base-specific cleavage and mass spectrometry.. Proc Natl Acad Sci U S A.

[29] Tobi EW, Lumey LH, Talens RP, Kremer D, Putter H (2009). DNA Methylation differences after exposure to prenatal famine are common and timing- and sex-specific.. Hum Mol Genet.

[30] Wang L, Wang F, Guan J, Le J, Wu L (2010). Relation between hypomethylation of long interspersed nucleotide elements and risk of neural tube defects.. Am J Clin Nutr.

[31] Lumey LH, Terry MB, Delgado-Cruzata L, Liao Y, Wang Q (2012). Adult global DNA methylation in relation to pre-natal nutrition.. Int J Epidemiol.

[32] Bennett ST, Lucassen AM, Gough SC, Powell EE, Undlien DE (1995). Susceptibility to human type 1 diabetes at IDDM2 is determined by tandem repeat variation at the insulin gene minisatellite locus.. Nat Genet.

[33] Coolen MW, Statham AL, Qu W, Campbell MJ, Henders AK (2011). Impact of the genome on the epigenome is manifested in DNA methylation patterns of imprinted regions in monozygotic and dizygotic twins.. PLoS One.

[34] Breton CV, Byun HM, Wenten M, Pan F, Yang A (2009). Prenatal tobacco smoke exposure affects global and gene-specific DNA methylation.. Am J Respir Crit Care Med.

[35] Hoyo C, Murtha AP, Schildkraut JM, Jirtle RL, Demark-Wahnefried W (2011). Methylation variation at IGF2 differentially methylated regions and maternal folic acid use before and during pregnancy.. Epigenetics.

[36] Waterland RA, Kellermayer R, Laritsky E, Rayco-Solon P, Harris RA (2010). Season of conception in rural gambia affects DNA methylation at putative human metastable epialleles.. PLoS Genet.

[37] Stoger R (2008). The thrifty epigenotype: an acquired and heritable predisposition for obesity and diabetes?. Bioessays.

[38] Murphy SK, Adigun A, Huang Z, Overcash F, Wang F (2012). Gender-specific methylation differences in relation to prenatal exposure to cigarette smoke.. Gene.

[39] Bouchard L, Thibault S, Guay SP, Santure M, Monpetit A (2010). Leptin gene epigenetic adaptation to impaired glucose metabolism during pregnancy.. Diabetes Care.

[40] Hoyo C, Fortner K, Murtha AP, Schildkraut JM, Soubry A (2012). Association of cord blood methylation fractions at imprinted insulin-like growth factor 2 (IGF2), plasma IGF2, and birth weight.. Cancer Causes Control.

[41] Talens RP, Jukema JW, Trompet S, Kremer D, Westendorp RG (2012). Hypermethylation at loci sensitive to the prenatal environment is associated with increased incidence of myocardial infarction.. Int J Epidemiol.

[42] Chotalia M, Smallwood SA, Ruf N, Dawson C, Lucifero D (2009). Transcription is required for establishment of germline methylation marks at imprinted genes.. Genes Dev.

[43] Waterland RA, Kellermayer R, Rached MT, Tatevian N, Gomes MV (2009). Epigenomic profiling indicates a role for DNA methylation in early postnatal liver development.. Hum Mol Genet.

[44] Breitling LP, Yang R, Korn B, Burwinkel B, Brenner H (2011). Tobacco-smoking-related differential DNA methylation: 27K discovery and replication.. Am J Hum Genet.

[45] Talens RP, Boomsma DI, Tobi EW, Kremer D, Jukema JW (2010). Variation, patterns, and temporal stability of DNA methylation: considerations for epigenetic epidemiology.. FASEB J.

[46] Waterland RA, Jirtle RL (2003). Transposable elements: targets for early nutritional effects on epigenetic gene regulation.. Mol Cell Biol.

[47] Morgan HD, Jin XL, Li A, Whitelaw E, O'Neill C (2008). The culture of zygotes to the blastocyst stage changes the postnatal expression of an epigentically labile allele, agouti viable yellow, in mice.. Biol Reprod.

[48] Heijmans BT, Tobi EW, Lumey LH, Slagboom PE (2009). The epigenome: archive of the prenatal environment.. Epigenetics.

[49] Heijmans BT, Mill J (2012). Commentary: The seven plagues of epigenetic epidemiology.. Int J Epidemiol.

[50] Zhang D, Cheng L, Badner JA, Chen C, Chen Q (2010). Genetic control of individual differences in gene-specific methylation in human brain.. Am J Hum Genet.

[51] Gibbs JR, van der Brug MP, Hernandez DG, Traynor BJ, Nalls MA (2010). Abundant quantitative trait loci exist for DNA methylation and gene expression in human brain.. PLoS Genet.

[52] Bell JT, Pai AA, Pickrell JK, Gaffney DJ, Pique-Regi R (2011). DNA methylation patterns associate with genetic and gene expression variation in HapMap cell lines.. Genome Biol.

[53] Bell CG, Finer S, Lindgren CM, Wilson GA, Rakyan VK (2010). Integrated genetic and epigenetic analysis identifies haplotype-specific methylation in the FTO type 2 diabetes and obesity susceptibility locus.. PLoS One.

[54] Nagaya K, Makita Y, Taketazu G, Okamoto T, Nakamura E (2009). Paternal allele of IGF2 gene haplotype CTG is associated with fetal and placental growth in Japanese.. Pediatr Res.

[55] Gaunt TR, Cooper JA, Miller GJ, Day IN, O'Dell SD (2001). Positive associations between single nucleotide polymorphisms in the IGF2 gene region and body mass index in adult males.. Hum Mol Genet.

[56] Heude B, Petry CJ, Pembrey M, Dunger DB, Ong KK (2006). The insulin gene variable number of tandem repeat: associations and interactions with childhood body fat mass and insulin secretion in normal children.. J Clin Endocrinol Metab.

[57] Bennett A, Sovio U, Ruokonen A, Martikainen H, Pouta A (2005). No association between insulin gene variation and adult metabolic phenotypes in a large Finnish birth cohort.. Diabetologia.

[58] Maas JA, Mook-Kanamori DO, Ay L, Steegers EA, van Duijn CM (2010). Insulin VNTR and IGF-1 promoter region polymorphisms are not associated with body composition in early childhood: the generation R study.. Horm Res Paediatr.

[59] D'Aleo V, Del GS, Groves C, Lupi R, Tancredi M (2011). INS VNTR class genotype and the function of isolated human islets.. Nutr Metab Cardiovasc Dis.

[60] Le SC, Fallin D, Schork NJ, Bougneres P (2000). The insulin gene VNTR is associated with fasting insulin levels and development of juvenile obesity.. Nat Genet.

[61] Davey SG, Leary S, Ness A, Lawlor DA (2009). Challenges and novel approaches in the epidemiological study of early life influences on later disease.. Adv Exp Med Biol.

[62] Petronis A (2010). Epigenetics as a unifying principle in the aetiology of complex traits and diseases.. Nature.

[63] Trienekens G, Smith DF, Phillips (2000). The food supply in the Netherlands during the Second World War.. Food, science, policy and regulation in the twentieth century. International and comparative perspectives.

[64] Burger GCE, Drummond JC, Sandstead HR (1948). Malnutrition and Starvation in Western Netherlands, September 1944–July 1945.

[65] Kent WJ, Sugnet CW, Furey TS, Roskin KM, Pringle TH (2002). The human genome browser at UCSC.. Genome Res.

[66] Tobi EW, Heijmans BT, Kremer D, Putter H, Delemarre-van de Waal HA (2011). DNA methylation of IGF2, GNASAS, INSIGF and LEP and being born small for gestational age.. Epigenetics.

[67] Li LC, Dahiya R (2002). MethPrimer: designing primers for methylation PCRs.. Bioinformatics.

[68] Coolen MW, Statham AL, Gardiner-Garden M, Clark SJ (2007). Genomic profiling of CpG methylation and allelic specificity using quantitative high-throughput mass spectrometry: critical evaluation and improvements.. Nucleic Acids Res.

[69] Thompson RF, Suzuki M, Lau KW, Greally JM (2009). A pipeline for the quantitative analysis of CG dinucleotide methylation using mass spectrometry.. Bioinformatics.

[70] Altshuler DM, Gibbs RA, Peltonen L, Altshuler DM, Gibbs RA (2010). Integrating common and rare genetic variation in diverse human populations.. Nature.

[71] Barrett JC, Fry B, Maller J, Daly MJ (2005). Haploview: analysis and visualization of LD and haplotype maps.. Bioinformatics.

[72] Adkins RM, Krushkal J, Klauser CK, Magann EF, Morrison JC (2008). Association between small for gestational age and paternally inherited 5′ insulin haplotypes.. Int J Obes (Lond).

[73] Adkins RM, Somes G, Morrison JC, Hill JB, Watson EM (2010). Association of Birth Weight with Polymorphisms in the IGF2, H19 and IGF2R Genes.. Pediatr Res.

[74] Petry CJ, Ong KK, Barratt BJ, Wingate D, Cordell HJ (2005). Common polymorphism in H19 associated with birthweight and cord blood IGF-II levels in humans.. BMC Genet.

[75] Petry CJ, Rayco-Solon P, Fulford AJ, Stead JD, Wingate DL (2009). Common polymorphic variation in the genetically diverse African insulin gene and its association with size at birth.. Hum Genet.

[76] Heude B, Ong KK, Luben R, Wareham NJ, Sandhu MS (2007). Study of association between common variation in the insulin-like growth factor 2 gene and indices of obesity and body size in middle-aged men and women.. J Clin Endocrinol Metab.

[77] O'Dell SD, Bujac SR, Miller GJ, Day IN (1999). Associations of IGF2 ApaI RFLP and INS VNTR class I allele size with obesity.. Eur J Hum Genet.

[78] Adkins RM, Fain JN, Krushkal J, Klauser CK, Magann EF (2007). Association between paternally inherited haplotypes upstream of the insulin gene and umbilical cord IGF-II levels.. Pediatr Res.

