# Supplementary material for: Prenatal Famine and Genetic Variation Are Independently and Additively Associated with DNA Methylation at Regulatory Loci within IGF2/H19
Source: PLoS One. 2012 May 30;7(5):e37933. doi: 10.1371/journal.pone.0037933 (PMC3364289; doi:10.1371/journal.pone.0037933)
Supplement: Table S1 — The primers and amplified regions. 1. The sequence of the forward primer, for Epityper a tag with the following sequence is added 5′: AGGAAGAGAG 2. The sequence of the reverse primer, for Epityper a tag with the following sequence is added 5′: CAGTAATACGACTCACTATAGGGAGAAGGCT PCR was performed with the following cycling protocol: 15 minutes at 95°C, 4 rounds of 20 seconds at 95°C, 30 seconds at 65°C, 1 minute at 72°C; followed by 40 rounds, 20 seconds at 95°C, 30 seconds at 58°C and 1 minute at 72°C; ending with 3 minutes at 72°C. (DOC) [file pone.0037933.s002.doc]

| **Locus** | **Location NCBI36/hg18** | **Strand** | **Amplicon (bp)** | **Forward primer^1^ 5’-3’** | **Reverse primer^2^ 5’-3’** |
| --- | --- | --- | --- | --- | --- |
| H19DMR | chr 11: 1975948-1976360 | - | 413 | GGGTTTGGGAGAGTTTGTGAGGT | ATACCTACTACTCCCTACCTACCAAC |
| IGF2DMR2 | chr11: 2111300-2111791 | + | 492 | GGAAAGGGGTTTAGGATTTTTAT | AACCACTCCCATTATAAACCTTTAAT |
| IGF2DMR2 CTCF | chr11 2112023-2112312 | + | 290 | TAGTAATGTTTAGTTGGAAGGGGAA | ACTACTTAACTCTAAAAACCCCTACCC |
| IGF2AS | chr11: 2117482-2117948 | + | 467 | TTTTAGAGAATTTAGGGGTTTTATTT | CCATACAAATAAAATTTAAACTATATTTCC |
| IGF2AS CTCF | chr11: 2118126-2118422 | - | 297 | GGTTGGAGGGTTTTAAAGTGG | AAAAAAACACTATAATTTTTACCAAATCAA |
| IGF2DMR0 upstr. | chr11: 2125961-2126065 | - | 105 | GTTGTGTGTTTAGTGGTTTTTGTTG | AAAAAATTTACCTAAAAAAAACTTCCC |
| IGF2DMR | chr11: 2126035-2126372 | - | 338 | TGGATAGGAGATTGAGGAGAAA | AAACCCCAACAAAAACCACT |
| IGF2DMR0 downstr. | chr11: 2127117-2127220 | - | 104 | GATGAGGTTTTTTTATTTGTAGGGG | AAAACCAAAATCCTAACAACTACCC |
| INSIGF | chr11:2138912-2139216 | - | 305 | GTTTTGAGGAAGAGGTGTTGA | ACCTAAAATCCAACCACCCTAA |
| LINES-1 | X58075: 335–767 | - | 432 | GTGTGAGGTGTTAGTGTGTTTTGTT | ATATCCCACACCTAACTCAAAAAAT |

Supplemental Table S1. The primers and amplified regions.
