# Supplementary material for: Prenatal Famine and Genetic Variation Are Independently and Additively Associated with DNA Methylation at Regulatory Loci within IGF2/H19
Source: PLoS One. 2012 May 30;7(5):e37933. doi: 10.1371/journal.pone.0037933 (PMC3364289; doi:10.1371/journal.pone.0037933)
Supplement: Table S2 — Information on individual CpG containing fragments. 1. CpG containing fragments (e.g. ‘CpG units’): excluded were fragments containing possible SNPs in CEU (by HAPMAP or 1000genomes), a measurement success rate below <75% or (partial) overlap with other units. 2. Mean methylation in %, based on the raw data. 3. the variation (in %) in the controls 4. The average within pair difference from a Linear Mixed Model, corrected for age and bisulfite batch. 5. The P value belonging to the within pair difference. (DOC) [file pone.0037933.s003.doc]

Supplemental Table S2. Information on individual CpG containing mass fragments.

| **Amplicon** | **CpGsite** | **Included^1^** | **Reason for exclusion** | **Success rate** | **Mean methylation^2^** | **SD^3^** | **Exp-Unexp^4^** | **P diff^5^** |
| --- | --- | --- | --- | --- | --- | --- | --- | --- |
| H19 DMR | CpG1 |  | Mass-overlap with fragment CpG 16 | 95.0 |  |  |  |  |
|  | CpG2 | Yes |  | 95.0 | 28.7 | 5.8 | -0.2 | 0.91 |
|  | CpG3.4.5 |  | Mass-overlap with fragment CpG 11 | 95.0 |  |  |  |  |
|  | CpG6 |  | low success rate | 65.8 |  |  |  |  |
|  | CpG7 | Yes |  | 89.2 | 32.2 | 3.2 | -0.7 | 0.23 |
|  | CpG8 | Yes |  | 94.2 | 32.1 | 2.0 | 0.0 | 0.99 |
|  | CpG9.10 | Yes |  | 94.2 | 26.8 | 2.6 | 0.0 | 0.99 |
|  | CpG11 |  | Mass-overlap with fragment CpG 3.4.5 | 95.8 |  |  |  |  |
|  | CpG12 |  | rs12292822 | 95.0 | 26.6 | 2.3 | 0.0 | 0.87 |
|  | CpG13 | Yes |  | 95.0 | 29.0 | 3.1 | -0.2 | 0.61 |
|  | CpG14.15 |  | rs12292818 | 95.0 | 31.5 | 2.5 | 0.0 | 0.98 |
|  | CpG16 |  | Mass-overlap with fragment CpG 1 | 95.0 |  |  |  |  |
|  | CpG17 |  | rs35592994 | 94.2 | 29.0 | 2.4 | -0.3 | 0.56 |
|  | CpG18.19 | Yes |  | 94.2 | 30.4 | 3.2 | -0.2 | 0.71 |
|  | CpG20 | Yes |  | 95.0 | 30.0 | 3.0 | -0.2 | 0.73 |
|  | CpG21 |  | Low success rate | 4.2 |  |  |  |  |
|  | CpG22 | Yes |  | 86.7 | 34.0 | 8.0 | -1.7 | 0.12 |
|  | CpG23 |  | Low success rate | 3.3 |  |  |  |  |
|  | CpG24 |  | Low success rate | 65.0 |  |  |  |  |
|  | CpG25 | Yes |  | 95.0 | 31.9 | 3.4 | -0.7 | 0.21 |
| IGF2 DMR2 | CpG1 |  | Low mass | 0.0 |  |  |  |  |
| S.L. | CpG2.3 |  | Mass-overlap with fragment CpG 9 | 65.8 |  |  |  |  |
| (DMR2) | CpG4 | Yes |  | 90.8 | 48.0 | 8.9 | 2.2 | 0.17 |
|  | CpG5.6 | Yes |  | 95.8 | 37.2 | 5.1 | -0.3 | 0.88 |
|  | CpG7 | Yes |  | 94.2 | 49.8 | 9.3 | 0.0 | 0.96 |
|  | CpG8 | Yes |  | 96.7 | 56.7 | 8.2 | 0.5 | 0.74 |
|  | CpG9 |  | Mass-overlap with fragment CpG 2.3 | 67.5 |  |  |  |  |
|  | CpG10 |  | Low mass | 0.0 |  |  |  |  |
|  | CpG11.12 | Yes |  | 95.0 | 47.7 | 5.6 | -0.2 | 0.91 |
|  | CpG13 | Yes |  | 93.3 | 44.3 | 7.4 | -0.3 | 0.83 |
|  | CpG14.15 | Yes |  | 96.7 | 55.5 | 8.2 | 0.7 | 0.68 |
|  | CpG16 |  | Mass-overlap with fragment CpG 18 and 21 | 95.8 |  |  |  |  |
|  | CpG17 |  | Low success rate | 3.3 |  |  |  |  |
|  | CpG18 |  | Mass-overlap with fragment CpG 16 and 21 | 95.8 |  |  |  |  |
|  | CpG19 |  | Low mass | 0.0 |  |  |  |  |
|  | CpG20 | Yes |  | 95.8 | 59.4 | 6.7 | -1.0 | 0.43 |
|  | CpG21 |  | Mass-overlap with fragment CpG 16 and 18 | 95.8 |  |  |  |  |
|  | CpG22 |  | High mass | 0.0 |  |  |  |  |
| IGF2 DMR2 CTCF | CpG1 | Yes |  | 95.0 | 53.9 | 3.8 | -1.7 | 0.040 |
| (DMR2) | CpG2 |  | Low success rate | 70.8 |  |  |  |  |
|  | CpG3 | Yes |  | 95.0 | 35.0 | 4.0 | -1.4 | 0.056 |
|  | CpG4 | Yes |  | 95.0 | 63.5 | 2.4 | -1.1 | 0.045 |
| IGF2AS | CpG1.2.3.4 |  | High mass | 0.0 |  |  |  |  |
| (DMR1) | CpG5 | Yes |  | 97.5 | 8.3 | 2.8 | 0.7 | 0.16 |
|  | CpG6 | Yes |  | 85.0 | 16.4 | 3.0 | 3.1 | 0.070 |
|  | CpG7 | Yes |  | 96.7 | 5.6 | 2.6 | -0.4 | 0.43 |
|  | CpG8.9.10 | Yes |  | 96.7 | 5.4 | 0.9 | -0.2 | 0.40 |
|  | CpG11.12 |  | High mass | 0.0 |  |  |  |  |
|  | CpG13.14.15.16 |  | High mass | 0.0 |  |  |  |  |
|  | CpG17 | Yes |  | 95.0 | 5.2 | 1.5 | 0.3 | 0.25 |
|  | CpG18 |  | Mass-overlap with fragment CpG 28 | 97.5 |  |  |  |  |
|  | CpG19.20.21 | Yes |  | 97.5 | 2.5 |  |  |  |
|  | CpG22 |  | Low success rate | 59.2 |  |  |  |  |
|  | CpG23.24.25.26.27 |  | High mass | 0.0 |  |  |  |  |
|  | CpG28 |  | Mass-overlap with fragment CpG 18 | 97.5 |  |  |  |  |
|  | CpG29 | Yes |  | 95.8 | 13.0 | 2.4 | 0.3 | 0.63 |
|  | CpG30 | Yes |  | 95.0 | 7.3 | 2.9 | 0.1 | 0.96 |
|  | CpG31.32.33 | Yes |  | 95.0 | 14.9 | 2.9 | 0.7 | 0.24 |
|  | CpG34.35.36.37 | Yes |  | 97.5 | 8.5 | 1.0 | 0.1 | 0.55 |
|  | CpG38 |  | Low mass | 0.0 |  |  |  |  |
|  | CpG39.40 | Yes |  | 81.7 | 11.7 | 2.2 | 0.5 | 0.43 |
|  | CpG41 | Yes |  | 97.5 | 3.1 | 0.8 | 0.5 | 0.0030 |
| IGF2AS CTCF | CpG1 | Yes |  | 98.3 | 2.5 | 1.7 | 0.2 | 0.61 |
| (DMR1) | CpG2 | Yes |  | 98.3 | 0.6 | 0.6 | 0.0 | 0.99 |
|  | CpG3.4 | Yes |  | 92.5 | 14.9 | 2.9 | 0.5 | 0.42 |
|  | CpG5.6.7.8 | Yes |  | 98.3 | 4.1 | 0.9 | 0.1 | 0.37 |
|  | CpG9.10 | Yes |  | 95.8 | 5.1 | 0.8 | 0.3 | 0.11 |
|  | CpG11.12 | Yes |  | 97.5 | 5.7 | 1.7 | 0.6 | 0.11 |
|  | CpG13.14.15.16 | Yes |  | 97.5 | 6.9 | 2.5 | 0.8 | 0.063 |
|  | CpG17.18.19 | Yes |  | 91.7 | 6.4 | 3.4 | 0.9 | 0.20 |
|  | CpG20 | Yes |  | 95.8 | 1.6 | 0.8 | 0.4 | 0.0054 |
|  | CpG21 |  | Low mass | 0.0 |  |  |  |  |
|  | CpG22 | Yes |  | 96.7 | 2.0 | 0.7 | 0.3 | 0.019 |
|  | CpG23.24 | Yes |  | 97.5 | 1.1 | 0.6 | 0.0 | 0.71 |
|  | CpG25.26.27 |  | High mass | 0.0 |  |  |  |  |
|  | CpG28.29.30.31 |  | High mass | 0.0 |  |  |  |  |
|  | CpG32 | Yes |  | 98.3 | 4.5 | 1.9 | 0.5 | 0.33 |
| IGF2 DMR0 upstream | CpG1 | Yes |  | 94.2 | 46.6 | 5.4 | -2.9 | 0.0056 |
| (DMR0) | CpG2 | Yes |  | 92.5 | 51.6 | 7.3 | -3.1 | 0.027 |
|  | CpG3 | Yes |  | 95.0 | 40.1 | 5.2 | -2.7 | 0.0056 |
|  | CpG4 | Yes |  | 95.8 | 50.4 | 4.0 | -1.4 | 0.049 |
|  | CpG5 | Yes |  | 91.7 | 32.6 | 4.3 | -0.5 | 0.57 |
| IGF2DMR0 | CpG1 |  | Low succes rate | 56.7 |  |  |  |  |
| downstream | CpG2 |  | Low succes rate | 67.5 |  |  |  |  |
| (DMR0) | CpG3 |  | Mass-overlap with fragment CpG 4 | 8.3 |  |  |  |  |
|  | CpG4 |  | Mass-overlap with fragment CpG 3 | 8.3 |  |  |  |  |
|  | CpG5.6 |  | Mass-overlap with fragment CpG 10 | 84.2 |  |  |  |  |
|  | CpG7 |  | Low succes rate | 44.2 |  |  |  |  |
|  | CpG8 | Yes |  | 77.5 | 82.7 | 5.7 | -2.3 | 0.05 |
|  | CpG9 | Yes |  | 75.8 | 60.6 | 5.8 | 1.0 | 0.35 |
|  | CpG10 |  | Mass-overlap with fragment CpG 5.6 | 82.5 |  |  |  |  |
|  | CpG11 |  | Low succes rate | 25.0 |  |  |  |  |
|  | CpG12.13 | Yes | rs11601832, but not present in CEU | 82.5 | 70.5 | 4.9 | -3.6 | 8.5E-4 |
| LINES-1 | CpG_1 | Yes |  | 98.3 | 64.2 | 2.6 | -1.8 | 5.8E-4 |
|  | CpG_2 | Yes |  | 98.3 | 60.0 | 1.4 | -0.8 | 0.004 |
|  | CpG_3 | Yes |  | 98.3 | 71.6 | 2.1 | -0.3 | 0.36 |
|  | CpG_4 | Yes |  | 96.7 | 36.9 | 4.6 | -0.4 | 0.28 |
|  | CpG_5 | Yes |  | 97.5 | 35.3 | 1.3 | -0.1 | 0.54 |
|  | CpG_6.7 | Yes |  | 98.3 | 69.4 | 2.0 | 0.1 | 0.13 |
|  | CpG_8.9 | Yes |  | 98.3 | 68.7 | 1.9 | 0.1 | 0.12 |
|  | CpG_10 |  | Low mass |  |  |  |  |  |
|  | CpG_11.12 | Yes |  | 98.3 | 83.6 | 2.5 | -0.4 | 0.31 |
