# Supplementary material for: Prenatal Famine and Genetic Variation Are Independently and Additively Associated with DNA Methylation at Regulatory Loci within IGF2/H19
Source: PLoS One. 2012 May 30;7(5):e37933. doi: 10.1371/journal.pone.0037933 (PMC3364289; doi:10.1371/journal.pone.0037933)
Supplement: Table S3 — The genotyping results for the H19 LD block. 1. Several SNPs were chosen from the HAPMAP CEU panel as tagging SNPs for the region, also several candidate SNPs were added. Some were both candidate as HAPMAP tagging SNPs. 2. Success rate of the genotyping. 3. Several SNPs could not be measured, one SNP had a low success rate and two SNPs were in perfect LD (r2>0.9) with another SNP in these individuals and thus not included in the final analysis. 4. The P value resulting from a test for Hardy-Weinberg disequilibrium, significant threshold is P<0.002 because of multiple testing. (DOC) [file pone.0037933.s004.doc]

Supplemental Table S3. The genotyping results for the H19 LD block

| **SNP** | **Source^1^** | **Success rate^2^** | **included?^3^** | **MAF(obs.)** | **MAF CEU** |  | **HW Pval^4^** | **associations (Pubmed [uid]: type)** |
| --- | --- | --- | --- | --- | --- | --- | --- | --- |
| rs217727 | both | 100 | YES | 0.204 | 0.15 | G:A | 0.16 | 15885138: birthweight and newborn  IGF2 levels |
| rs2839701 | tagging | no design poss. | |  |  |  |  |  |
| rs2067051 | both | 94.2 | No, below <95% | 0.5 | 0.482 | C:C | 0.89 | 20639793: association with birth weight |
| rs2251375 | both | 98.3 | YES | 0.297 | 0.292 | C:A | 0.74 | 20639793: association with birth weight |
| rs10732516 | candidate | 98.3 | covered by rs4929983 | 0.496 | NA, 0.44 in Brazilians | T:C | 1 | In core binding motif 6^th^ CTCF ICR |
| rs11042170 | tagging | no design poss. | |  |  |  |  |  |
| rs2735971 | tagging | no design poss. | |  |  |  |  |  |
| rs12417375 | tagging | no design poss. | |  |  |  |  |  |
| rs4929983 | tagging | 100 | YES | 0.488 | 0.397 | T:C | 0.74 |  |
| rs4929984 | both | 98.3 | covered by rs4929983 | 0.47 | 0.486 | A:C | 0.86 | 20639793: association with birth weight |
| rs12292757 | tagging | 98.3 | YES | 0.212 | 0.125 | G:A | 0.47 |  |
