# Supplementary material for: Prenatal Famine and Genetic Variation Are Independently and Additively Associated with DNA Methylation at Regulatory Loci within IGF2/H19
Source: PLoS One. 2012 May 30;7(5):e37933. doi: 10.1371/journal.pone.0037933 (PMC3364289; doi:10.1371/journal.pone.0037933)
Supplement: Table S4 — The genotyping results for the INSIGF LD blocks. 1. Several SNPs were chosen from the HAPMAP CEU panel as tagging SNPs for the region, also several candidate SNPs were added. Some were both candidate as HAPMAP tagging SNPs. 2. Success rate of the genotyping. 3. Several SNPs could not be measured, one SNP had a low success rate and two SNPs were in perfect LD (r2>0.9) with another SNP in these individuals and thus not included in the final analysis. 4. The P value resulting from a test for Hardy-Weinberg disequilibrium, significant threshold is P<0.002 because of multiple testing. (DOC) [file pone.0037933.s005.doc]

Supplemental Table S4. The genotyping results for the INSIGF LD blocks

| **SNP** | **Source^1^** | **Success rate^2^** | **included?^3^** | **MAF(obs.)** | **MAF CEU** |  | **HW Pval^4^** | **associations (Pubmed [uid]: type)** |
| --- | --- | --- | --- | --- | --- | --- | --- | --- |
| rs11042594 | tagging | 98.3 | no, covered by rs680  (r^2=0.90) | 0.305 | 0.341 | G:A | 0.046 | - |
| rs10840356 | tagging | 0 | below <95% | - | - | - | - | - |
| rs4341514 | tagging | 100 | out of HW P<0.002 | 0.471 | 0.442 | T:C | 3.14E-21 | - |
| rs7873 | tagging | 100 | YES | 0.079 | 0.102 | A:G | 0.92 | - |
| rs3802971 | tagging | 100 | YES | 0.092 | 0.099 | C:T | 0.69 | - |
| rs680 | candidate | 100 | YES | 0.321 | 0.33 CEU 1000genomes | G:A | 0.076 | 11448941: BMI adult men, 17289909: muscle functioning, 19434426: birth length, 10573016: body weight in men |
| rs3213223 | candidate | 100 | YES | 0.238 | 0.199 | C:T | 1.0 | - |
| rs3213221 | both | 100 | YES | 0.412 | 0.434 | C:G | 0.11 | 17289909 :loss of strength following excercise, 17339271:association with IGF2 DMR methylation |
| rs3213216 | tagging | 99.2 | no variance | 0 | 0.345 | G:G | 1.0 | - |
| rs3741212 | tagging | no design poss. | | - | - | - | - | - |
| rs11603378 | tagging | 0 | below <95% | - | - | - | - | - |
| rs1003483 | both | 100 | YES | 0.424 | 0.46 | T:G | 0.0399 | 19390492: no association CTCF6 and H19DMR methylation, marginal association with paternal haplotype and SGA and placental growth, 17339271: IGF2 DMR methylation |
| rs1003484 | candidate | 100 | covered by rs2239681  (r^2=1.0) | 0.3 | CEU 0.25 1000genomes | G:A | 0.0035 | 17339271:methylation IGF2DMR |
| rs2239681 | tagging | 100 | YES | 0.3 | 0.27 | G:A | 0.0035 | - |
| rs3741211 | both | 100 | YES | 0.374 | 0.389 | A:G | 0.0063 | 19546867: association IGF1BP1 levels, 21078522: endometrial cancer risk, 11448941: adult BMI, 17488802: adult height; 19390492: no association with CTCF6 and H19DMR methylation, marginal association with paternal haplotype transmission and SGA and placental growth |
| rs3741209 | candidate | 100 | covered by rs3741211  (r^2=1.0) | 0.379 | 0.375 | G:C | 0.0043 | 18955703: abolishes CpG site in IGF2 DMR |
| rs3741206 | tagging | no design poss. | - | - | - | - | - |  |
| rs4320932 | tagging | 100 | out of HW P<0.002 | 0.238 | 0.204 | T:C | 9.00E-04 | - |
| rs10840442 | tagging | no design poss. | - | - | - | - | - |  |
| rs7924316 | both | 100 | YES | 0.438 | 0.465 | T:G | 0.32 | 17289909: strength loss following excercise |
| rs10840447 | tagging | 100 | YES | 0.392 | 0.376 | C:T | 0.017 | - |
| rs3842756 | tagging | 100 | YES | 0.292 | 0.243 | G:A | 0.53 | 12610512: prostate cancer risk |
| rs689 | candidate | 100 | YES | 0.367 | 0.242 | A:T | 0.85 | 19434426: postnatal growth, 16608900: BMI in children, 17667841: paternal transmission associates with newborn IGF2 levels, 17700581: association with SGA risk, 15047631: head circumference at birth, newborn IGF2 levels, 10573016: body weight in men, 11101842: T2D, 11528401: paternal transmission with child BMI and insulin secretion, 9590300: birth size |
| rs3842738 | candidate | 100 | no variance | 0 | 0.0 CEU 1000genome | C:C | 1 | 17667841: paternal haplotype transmission associates with newborn IGF2 levels, 17700581: paternal haplotype transmission associates with SGA risk |
