# Supplementary material for: Prenatal Famine and Genetic Variation Are Independently and Additively Associated with DNA Methylation at Regulatory Loci within IGF2/H19
Source: PLoS One. 2012 May 30;7(5):e37933. doi: 10.1371/journal.pone.0037933 (PMC3364289; doi:10.1371/journal.pone.0037933)
Supplement: Table S7 — The effect of the tagging SNPs on DNA methylation. The effect of the rare allele on DNA methylation, assuming an additive model. The beta is the change in average DNA methylation (%). For instance, a beta of 1.0 means that each allele increases the amount of DNA methylation at that DMR with 1.0%. The P value is given followed by the P value corrected for multiple testing (FDR). (DOC) [file pone.0037933.s008.doc]

Table S7. The effect of the tagging SNPs on DNA methylation

| **IGF2 DMR0** | **beta** | **SD** | **P** | **P_BH_** |
| --- | --- | --- | --- | --- |
| rs2251375 | -0.4 | -0.1 | 0.41 | 0.87 |
| rs217727 | 0.0 | 0.0 | 0.99 | 0.99 |
| rs4929983 | -0.2 | -0.1 | 0.61 | 0.87 |
| rs12292757 | 0.3 | 0.1 | 0.56 | 0.87 |
| rs7873 | -0.6 | -0.2 | 0.48 | 0.87 |
| rs3802971 | 1.6 | 0.5 | 0.036 | 0.23 |
| rs680 | -1.1 | -0.3 | 0.017 | 0.17 |
| rs3213223 | 1.0 | 0.3 | 0.047 | 0.27 |
| rs1003483 | 0.6 | 0.2 | 0.14 | 0.53 |
| rs2239681 | -1.3 | -0.4 | 0.0011 | 0.027 |
| rs3213221 | -0.6 | -0.2 | 0.13 | 0.53 |
| rs3741211 | 0.9 | 0.3 | 0.022 | 0.18 |
| rs7924316 | 0.3 | 0.1 | 0.50 | 0.87 |
| rs10840447 | 0.5 | 0.1 | 0.26 | 0.85 |
| rs3842756 | 0.4 | 0.1 | 0.34 | 0.87 |
| rs689 | 0.7 | 0.2 | 0.11 | 0.53 |
| **INSIGF** | **beta** | **SD** | **P** | **P_BH_** |
| rs2251375 | 0.6 | 0.2 | 0.29 | 0.87 |
| rs217727 | 0.4 | 0.1 | 0.52 | 0.87 |
| rs4929983 | -0.4 | -0.2 | 0.33 | 0.87 |
| rs12292757 | -1.2 | -0.5 | 0.018 | 0.17 |
| rs7873 | 0.5 | 0.2 | 0.58 | 0.87 |
| rs3802971 | -0.4 | -0.2 | 0.60 | 0.87 |
| rs680 | 0.4 | 0.1 | 0.44 | 0.87 |
| rs3213223 | -1.2 | -0.4 | 0.021 | 0.18 |
| rs1003483 | 0.3 | 0.1 | 0.40 | 0.87 |
| rs2239681 | 1.1 | 0.4 | 0.0092 | 0.13 |
| rs3213221 | 0.1 | 0.0 | 0.81 | 0.95 |
| rs3741211 | -1.5 | -0.6 | 1.1E-04 | 0.0034 |
| rs7924316 | 0.9 | 0.3 | 0.031 | 0.21 |
| rs10840447 | 0.1 | 0.0 | 0.79 | 0.95 |
| rs3842756 | -2.0 | -0.8 | 8.2E-06 | 0.00039 |
| rs689 | -2.3 | -0.9 | 7.4E-08 | 7.10E-06 |
