# Supplementary material for: Prenatal Famine and Genetic Variation Are Independently and Additively Associated with DNA Methylation at Regulatory Loci within IGF2/H19
Source: PLoS One. 2012 May 30;7(5):e37933. doi: 10.1371/journal.pone.0037933 (PMC3364289; doi:10.1371/journal.pone.0037933)
Supplement: Table S8 — Famine associations corrected for significant SNPs. For each locus the beta for the association with famine is given, now corrected for the SNPs showing (nominally) significant associations with this locus, followed by the corrected P-value. (DOC) [file pone.0037933.s009.doc]

Supplemental Table S8. Famine associations corrected for significant SNPs

| **DMR** | **Corrected within pair diff. (%)** | **P SNP corrected** |
| --- | --- | --- |
| *H19* DMR | No SNPs | No SNPs |
| *IGF2* DMR S.L. | 0.4 | 0.78 |
| *IGF2* DMR CTCF | No SNPs | No SNPs |
| *IGF2* DMR1 | 0.3 | 0.028 |
| *IGF2* DMR0 | -1.9 | 6.8x10^-6^ |
| *INSIGF* | -1.2 | 0.027 |
