# Supplementary material for: Prenatal Famine and Genetic Variation Are Independently and Additively Associated with DNA Methylation at Regulatory Loci within IGF2/H19
Source: PLoS One. 2012 May 30;7(5):e37933. doi: 10.1371/journal.pone.0037933 (PMC3364289; doi:10.1371/journal.pone.0037933)
Supplement: Table S9 — SNP associations with and without famine exposure correction. The first column denotes the SNP- DNA methylation locus under investigation. Column two and three contain the beta and resulting P-value of the SNP-DNA methylation association corrected for famine exposure, the fourth and fifth the beta and P-value without famine correction. There is no notable difference between the two models. (DOC) [file pone.0037933.s010.doc]

Supplemental Table S9. SNP associations with and without famine exposure correction

| **Association between** | **with famine exposure** | | **without famine exposure** | |
| --- | --- | --- | --- | --- |
| ***DMR* – SNP** | **beta** | **P** | **beta** | **P** |
| *IGF2* DMR0-rs2239681 | -1.3 | 1.1x10^-3^ | -1.4 | 9.9x10^-4^ |
| *INSIGF*-rs3842756 | -2.0 | 8.2x10^-6^ | -2.1 | 1.4x10^-5^ |
| *INSIGF*-rs689 | -2.3 | 7.4x10^-8^ | -2.4 | 4.0x10^-8^ |
