# Supplementary material for: Prenatal Famine and Genetic Variation Are Independently and Additively Associated with DNA Methylation at Regulatory Loci within IGF2/H19
Source: PLoS One. 2012 May 30;7(5):e37933. doi: 10.1371/journal.pone.0037933 (PMC3364289; doi:10.1371/journal.pone.0037933)
Supplement: Table S10 — Nine out of sixteen tagging SNPs were CpG altering polymorphisms. (DOC) [file pone.0037933.s011.doc]

Supplemental Table S10. Nine out of sixteen tagging SNPs were CpG altering polymorphisms

| SNP | CpG SNP? |
| --- | --- |
| rs2251375 | No |
| rs217727 | Yes |
| rs4929983 | Yes |
| rs12292757 | Yes |
| rs7873 | Yes |
| rs3802971 | Yes |
| rs680 | Yes |
| rs3213223 | Yes |
| rs1003483 | No |
| rs2239681 | Yes |
| rs3213221 | No |
| rs3741211 | No |
| rs7924316 | No |
| rs10840447 | Yes |
| rs3842756 | No |
| rs689 | No |
